# Combining Protein Expression and Molecular Data Improves Mutation Characterization of Dystrophinopathies

**DOI:** 10.3389/fneur.2021.718396

**Published:** 2021-12-07

**Authors:** Gisela Gaina, Rolf H. A. M. Vossen, Emilia Manole, Doina Anca Plesca, Elena Ionica

**Affiliations:** ^1^Department of Biochemistry and Molecular Biology, University of Bucharest, Bucharest, Romania; ^2^Laboratory of Cell Biology, Neuroscience and Experimental Myology, Victor Babes National Institute of Pathology, Bucharest, Romania; ^3^Center for Human and Clinical Genetics, Leiden Genome Technology Center, Leiden, Netherlands; ^4^Colentina Clinical Hospital, Bucharest, Romania; ^5^Department of Pediatrics, Carol Davila University of Medicine and Pharmacy, Bucharest, Romania; ^6^Department of Clinical Pediatrics, Victor Gomoiu Children Clinical Hospital, Bucharest, Romania

**Keywords:** Duchenne/Becker muscular dystrophy (DMD/BMD), dystrophin, *DMD* gene, immunofluorescence, multiplex western blot, MLPA, HRM analysis

## Abstract

Duchenne and Becker muscular dystrophy are X-linked recessive inherited disorders characterized by progressive weakness due to skeletal muscle degeneration. Different mutations in the *DMD* gene, which encodes for dystrophin protein, are responsible for these disorders. The aim of our study was to investigate the relationship between type, size, and location of the mutation that occurs in the *DMD* gene and their effect on dystrophin protein expression in a cohort of 40 male dystrophinopathy patients and nine females, possible carriers. We evaluated the expression of dystrophin by immunofluorescence and immunoblotting. The mutational spectrum of the *DMD* gene was established by MLPA for large copy number variants, followed by HRM analysis for point mutations and sequencing of samples with an abnormal melting profile. MLPA revealed 30 deletions (75%) and three duplications (7.5%). HRM analysis accounted for seven-point mutations (17.5%). We also report four novel small mutations (c. 8507G>T, c.3021delG, c.9563_9563+1insAGCATGTTTATGATACAGCA, c.7661-60T>A) in *DMD* gene. Our work shows that the DNA translational open reading frame and the location of the mutation both influence the expression of dystrophin and disease severity phenotype. The proposed algorithm used in this study demonstrates its accuracy for the characterization of dystrophinopathy patients.

## Introduction

The dystrophinopathies, Duchenne muscular dystrophy (DMD), and Becker muscular dystrophy (BMD) represent a group of genetic conditions characterized by progressive symmetrical muscle weakness and wasting (1). Both are allelic disorders with similar signs, symptoms, and patterns of muscle involvement with a difference in severity, age of onset, and rate of disease progression. DMD (OMIM #310200), one of the most common and severe forms of muscular dystrophy has a worldwide incidence of one in 3,500 live male births (1, 2), and with onset in childhood and death in early adulthood. The allelic form BMD (BMD; OMIM #300376) has an estimated incidence of one in 18,000 males, with later onset, a slower rate of progression, and an average life expectancy of around 40 years (2). Dystrophinopathies are caused by X-linked recessive mutations in the dystrophin gene (*DMD*) (*DMD*, MIM#300377). This gene is the largest known human gene spanning more than 2.2 Mb of genomic DNA, located on chromosome Xp21.2 and consisting of 79 exons which form a 14-kb mRNA transcript (3, 4). The product of the gene is a 427 kDa (kilodalton) sarcolemmal protein called—dystrophin—(OMIM 300377) which represents 0.002% of the total striated muscle protein (5). Dystrophin is composed of 3,685 amino acids (aa) and organized into four distinct structural domains (5, 6): (I) the N-terminal actin-binding domain (aa 12–240), (II) the central rod domain encoding 24 spectrin repeats, four hinges regions, which contains a binding region for neuronal nitric oxide synthesis (nNOS) (aa 253–3,112) (7), (III) the cysteine-rich domain (aa 3,113–3,299) important for binding dystrophin to the β-dystroglycan (8–10), and (IV) the carboxy-terminal (aa 3,300–3,685) that binds dystrophin to the glycoprotein complex (11, 12).

Previously reported mutations that affect structure and protein expression in the *DMD* gene include deletions of one or more exons as seen in ~60–65% of DMD patients (13, 14) and 85% of BMD patients (15). Duplications occur in 5–8% of patients for both DMD and BMD (16, 17), and small mutations occur in 30–35% of patients (18). Various large mutations in the *DMD* gene tend to cluster within two hot-spot regions with small differences within the population: between exons 2–20, mutations that remove some or all the actin-binding sites together with a part of the rod domain (19) and between exons 44–55 mutation that remove a part of the rod domain necessary for correct localization of nNOS to the sarcolemma (20). Small mutations can occur anywhere in the gene (18) and are usually detected by the sequencing of all exons.

One explanation for the difference in disease severity between DMD and BMD phenotypes lies in the differences in dystrophin expression caused by a large variety of mutations in the *DMD* gene (13). It has been suggested that the severity of the phenotype depends on whether or not the mutation disturbs the reading frame. According to the theory proposed by Monaco in 1988 (14), mutations that maintain the reading frame encode a partially functional protein and lead to a BMD phenotype, while mutations that disrupt the reading frame lead to the complete or near-complete absence of dystrophin protein are associated with a DMD phenotype (15). The reading frame hypothesis has been reported to be correct in around 90% of DMD/BMD cases. However, many exceptions from the reading frame rule have been reported over time (16, 17). This means that the complexity of the diseases is much greater, and it is not only the reading frame that influences the dystrophin expression, but also mutation location, type, and size.

To understand more about the genotype-phenotype correlations and to shed light on the pathogenic mechanism of dystrophinopathies, we describe our analysis strategy of type, size, and location of mutations in the *DMD* gene by three molecular methods: (a) multiplex ligation-dependent probe amplification (MLPA) assay, a highly sensitive method able to identify the presence of large mutations (deletions/duplications) in a gene, (b) high resolution melting curve (HRM) analysis for screening of small/point mutations, (c) sequencing of samples with abnormal melting profiles in 40 Romanian DMD/BMD male patients and nine females possible carriers. Screening with immunofluorescence (IF) and multiplex western blot (WB) helped to a complete molecular characterization of patients with dystrophinopathy enabling us to explore the correlation between mutation and the effect on protein expression, which are essential for the application of the newest specific molecular therapeutic strategies.

## Materials and Methods

### Sample Workflow and Patient Selection

A cohort of 180 patients with a clinical diagnosis of muscular dystrophy was referred to our department for muscle protein analysis. The clinical diagnosis took placed at the Clinical Hospital “Victor Gomoiu,” Bucharest, and was based on a neurological evaluation, creatine phosphokinase (CPK) level, and a typical myopathic pattern on an electromyography test (EMG). The muscle biopsy taken from gastrocnemius muscle and routine histological examination of the muscular tissue were performed at Colentina Clinical Hospital, Bucharest.

Although recent advances in molecular genetics research resulted in the development of less invasive diagnostic methods, at the time of the study, the genetic tests were not available for Romanian patients. Therefore, muscle biopsy analysis was the first step in the diagnosis, and it is still an important tool for patients with different forms of muscular dystrophy, for which genetic tests are not currently implemented in diagnosis. Molecular tests were performed in the Center for Human and Clinical Genetics, Leiden (the Netherlands).

**Figure 1:**
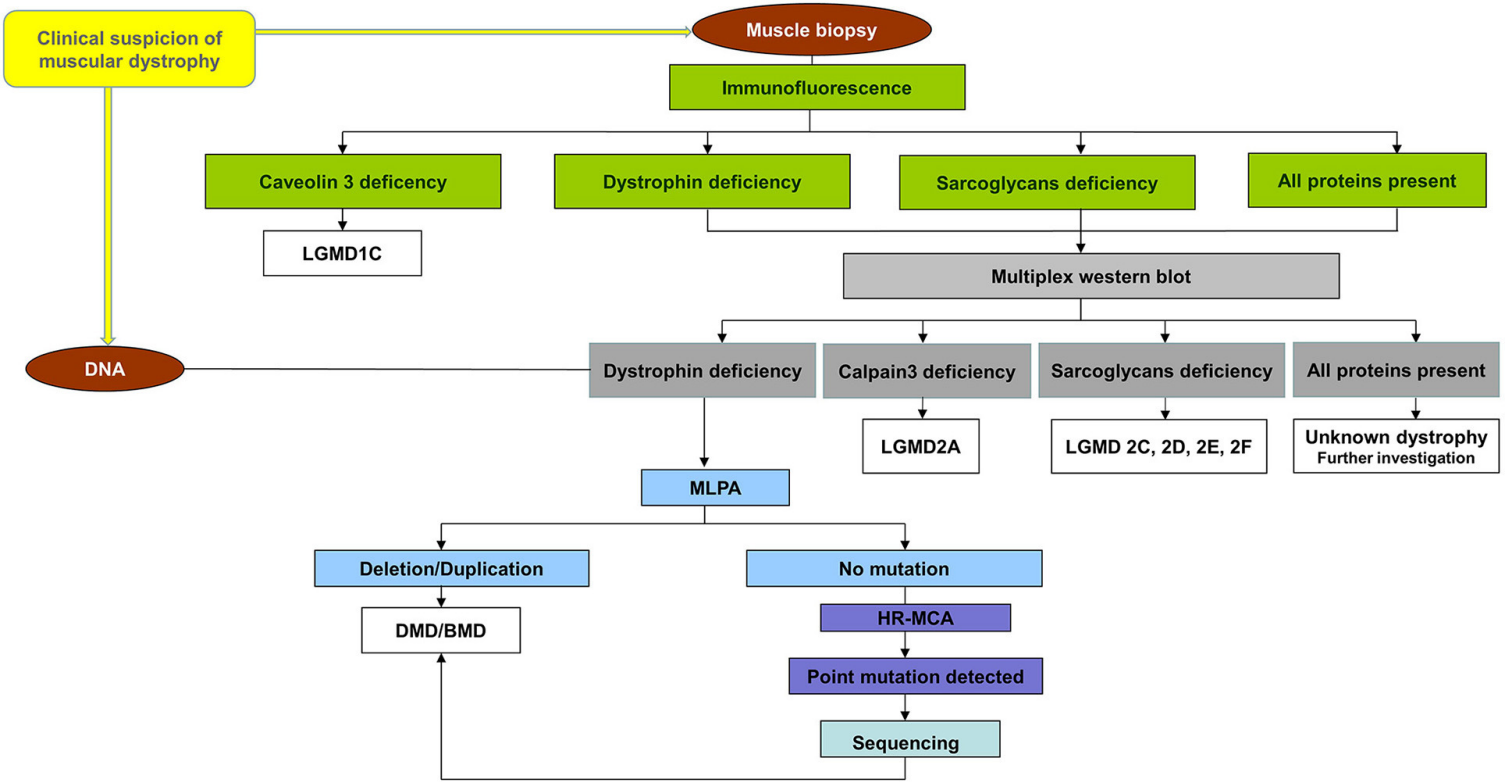
Workflow chart for all patients referring to us. All patients were first immunohistochemical analyzed using a panel of antibodies for muscle proteins such as dystrophin, utrophin, α, β, γ, sarcoglycans caveolin-3, dysferlin, merosin, and nNOS to establish the primary protein deficiency. Based on the results obtained by immunohistochemical staining, the patients were classified into four groups: (a) patients with a deficiency of dystrophin, (b) patients with a deficiency of sarcoglycans, (c) patients with a deficiency of caveolin 3, and (d) patients with all proteins present (data not shown). Patients from the group (a), (b), and (d) were further analyzed by multiplex WB to confirm the immunohistochemical result and to identify modification of calpain-3 for the others. A total of 40 unrelated male patients identified with a deficiency of dystrophin were recruited for this study. These patients were further tested for a complete molecular characterization of the *DMD* gene mutations.

The participants who enrolled in the project followed the workflow chart shown in Figure 1. A total of 40 unrelated male patients identified with a deficiency of dystrophin were recruited for this study.

The group of females (*n* = 9) included in the study comprised symptomatic female patients (*n* = 4) and asymptomatic females (*n* = 5). The symptomatic female patients comprised two dizygotic twins, and two girls three and four years. The muscle biopsy was available only for the twin sisters. The asymptomatic females were relatives of the affected patients (*n* = 5): mother, sister, and two primary cousins of patient #1 and the twin's mother. They were voluntarily enrolled in this study for molecular analysis. No muscle biopsies were available, and no clinical examinations were performed.

To identify the spectrum of mutations in the *DMD* gene, we undertook a systematic investigation of DNA from all 40 patients with a dystrophin deficiency as well as from an additionally female group. Three different methods were used: MLPA method for large copy number variants (deletions/duplications), followed by HRM analysis of samples with no large mutations found, and sequencing for samples with a modification of the melting curve.

As a normal control, samples (muscle biopsy and blood) from patients without any muscle disease were used.

The study was approved by the ethics committees of the institutions by a contractual agreement between them. Every family was informed about the study and informed consent was obtained from each patient/parent.

### Muscle Protein Analysis

The dystrophin expression was evaluated by two main complementary techniques, immunofluorescence (IF) and immunoblotting (WB).

#### Immunohistochemical Analysis

Muscular cryosections (7 um thick) were taken from male patients, dizygotic twins, and controls. They were processed for indirect immunofluorescence according to standard procedure. Immunostaining for dystrophin was performed using monoclonal antibodies against three domains: rod-domain (NCL-DYS1-clone Dy4/6D3), C-terminus domain (NCL-DYS2 clone Dy8/6C5) and N-terminus domain (NCL-DYS3 clone Dy10/12B2; Novocastra Laboratories Ltd, Newcastle upon Tyne, UK). All antibodies were diluted in 2% BSA (Bovine Serum Albumine) in PBS (Phosphate Buffered Saline). The secondary antibody was a biotinylated goat anti-mouse antibody IgG (SC- 53799, Santa Cruz Biotechnology, CA). The fluorescent signal was revealed using Streptavidin-FITC from Streptomyces avidinii (Sigma-Aldrich, USA). The protein expression was evaluated with an Olympus fluorescent microscope.

#### Immunoblot Analysis

The multiplex WB analysis was performed as previously described (21) with some modifications (22). After SDS-PAGE gel electrophoresis and semi-dry transfer of proteins on a nitrocellulose membrane, the visualization of corresponding bands was done by a chromogenic method with WesternBreeze/^®^Chromogenic Western Blot Immunodetection Kit, (Invitrogen, USA) using the same antibodies as in the IF method. Photographs of all blots were taken with the Vilber Lourmat System (Bio-Profil, Germany). The bands corresponding to myosin heavy chains (MHC) in the post-transfer-stained gel demonstrate equal protein loading on each lane.

In the controls and BMD patients, the dystrophin level was determined by densitometry of dystrophin bands using ImageJ software. For protein data, myosin bands normalization in the post-transfer, Coomassie blue-stained were used.

### Blood Samples and Molecular Studies

Genomic DNA was isolated from 3 ml venous blood harvested on EDTA anticoagulant using the Wizard^®^ Genomic DNA Purification kit Promega (Madison, USA) according to the manufacturer's recommendations. The quantity and quality of the DNA were spectrophotometrically determined.

#### MLPA Analysis

For large genomic rearrangement investigations in the *DMD* gene, the MLPA method was performed according to the instructions of the manufacturer. SALSA MLPA kit P034/P035DMD/Becker, available at MRC-Holland (Amsterdam, NL) was used. Fragment analysis was performed with GeneMapper ID v3.1 software.

#### HRM Analysis

Samples without deletions or duplications in the *DMD* gene by MLPA (*n* = 7) and samples from the female group (*n* = 9) were screened for point mutations. This technology previously described (23), involved a PCR amplification of the DNA fragments of interest with a specific pair of M13-tailed primers in the presence of a fluorescent binding dye (LCGreen), (Idaho Technology, USA) and a high-resolution melting instrument LightScanner^®^ System (Idaho Tech, USA). PCR amplification was followed by the fluorescence measurement of PCR products when amplicons are gradually denatured by increasing the temperature to 94°C then cooling to 40°C to produce a specific melting profile. The presence of a mutation in PCR products determines a modification in the shape of the DNA melting curves compared with the melting profile of the wild-type (normal) DNA. Interpretation of the results was made with LightScanner Software.

#### Sequencing

The Sanger DNA sequencing method was applied to identify the subtle mutations in samples that showed a modification of the melting curve by HRM on sequencer analyser ABI PRISM 3130 (Applied Biosystems, Foster City, CA, USA). The sequence of the primers and the specific annealing temperatures for PCR were obtained from the Leiden Muscular Dystrophy website (www.dmd.nl)^1^, and Center for Human and Clinical Genetics. The resultant sequences were analyzed by alignment with standard sequences of the *Homo sapiens DMD* gene (NM_004006.2.) from GenBank (Bethesda, MD, USA; www.ncbi.nlm.nih.gov) using the BioEdit software.

### Variant Analysis

Prediction of the pathogenicity of all variants identified from sequencing was performed with Alamut Visual software. The functional impact of amino acid changes was predicted by PolyPhen-2 (http://genetics.bwh.harvard.edu/pph2). Based on the prediction of these two software programs and follow the ACMG guidelines, the variants identified were classified as pathogenic, likely pathogenic, benign, likely benign, or variants of unknown significance.

### Statistical Analysis

The data obtained data were analyzed using statistical software GraphPad Prism 5.0 software.

## Results

The baseline characteristics of the male 40 patients with a deficiency of dystrophin are shown in Table 1. The patient numbers used in the text refer to this table.

**Table 1:** The baseline characteristics of the study male cohort.

**Case**	**Age at biopsy**	**CPK U/I**	**IF**			**WB**		**MLPA**	**Mutation type**	**Phenotype**
			**NCl-Dys 1**	**NCL-Dys 2**	**NCL-Dys 3**	**NCL-Dys 1**	**NCL-Dys 2**			
1	3.5	57,660	-	-	-	-	-	Del 48–50	OF	DMD
2	6	2,099	-	-	-	-	-	Del 48–50	OF	DMD
3	3	NA	+	+/−	+	-	-	Del 48–50	OF	DMD
4	8	5,400	+/−	+/−	+/−	-	-	Del 48–50	OF	DMD
5	6	NA	+/−	+/−	-	-	-	Del 48–50	OF	DMD
6	6	1,313	-	-	-	-	-	Del 3–21	OF	DMD
7	7	1,730	-	+/−	+/−	+/−	/-	Del 53–62	OF	BMD ±
8	7	4,500	+/−	+/−	+/−	-	+/−	Del 45–53	IF	BMD
9	8	1,010	+/−	+/−	+/−	-	-	Del 51	OF	DMD
10	4	NA	+/−	-	-	-	-	Del 49–53	IF	DMD ±
11	6	1,500	+/−	+/−	+/−	-	-	Del 46–47	OF	DMD
12	5	NA	-	-	-	-	-	Del 46–47	OF	DMD
13	9	16,924	-	-	-	-	-	Del 45–50	OF	DMD
14	7	13,521	-	-	-	-	-	Del 45–50	OF	DMD
15	11	6,500	-	-	-	-	-	Del 45–50	OF	DMD
16	6	16,110	+/−	+/−	+/−	-	-	Del 45–50	OF	DMD
17	4	9,490	+	+	+	+/−	+/−	Del 48–49	IF	BMD
18	5	NA	+/−	+/−	+/−	-	-	No deletion, No duplication		DMD
19	0,5	2,752	-	-	-	-	-	No deletion, No duplication		DMD
20	12	2,231	+/−	+/−	+-	+/−	-	Del 48–49	IF	BMD
21	2	23,500	-	+/−	+/−	-	-	No deletion, No duplication		DMD
22	4	8,799	-	-	-	-	-	Dup 14–17	OF	DMD
23	7	NA	-	+/−	+/−	+/−	+/−	Dup 61–78	IF	BMD
24	17	1,050	+	+	+	+/−	+/−	Del 45–47	IF	BMD
25	7	942	-	-	-	-	-	Del 45	OF	DMD
26	4	2,468	+/−	+/−	+/−	-	-	Del 46–50	OF	DMD
27	0.4	12,200	-	-	-	-	-	Del 49–50	OF	DMD
28	6	9,877	-	-	-	-	-	No deletion, No duplication		DMD
29	3	251	+/−	+/−	+/−	+/−	+/−	Del 45–47	IF	BMD
30	12	1,080	+	+	+	+/−	+/−	Del 45–47	IF	BMD
31	9	10,600	-	-	+/−	-	-	Del 46–48	OF	DMD
32	8	NA	-	-	+/−	-	-	Del 46–48	OF	DMD
33	10	10,760	+/−	+	+	-	-	No deletion, No duplication		DMD
34	5	21,000	-	-	-	-	-	No deletion, No duplication		DMD
35	9	NA	+	+	+	+/−	+	Del 48–49	IF	BMD
36	8	1,500	-	-	+/−	-	-	Del 48–52	OF	DMD
37	4	N	+/−	+/−	-	-	-	No deletion, No duplication		DMD
38	2	NA	+/−	+	+/−	+/−	-	Del 3–30	IF	BMD
39	2	4,000	-	+/−	-	-	-	Del 46–52	OF	DMD
40	12	4,267	-	-	-	-	-	Dup 17–21	OF	DMD

*Del, deletion; Dup, duplication; N, normal; NA, not available; DMD, Duchenne Muscular Dystrophy; BMD, Becker Muscular Dystrophy. ± Exceptions to the reading frame rule*.

### Clinical and Histopathological Characteristics

The age of DMD patients at the time of muscle biopsy ranged from 4 months to 17 years. With one exception (patient #37, who presented normal level of CPK), all patients showed elevated serum CPK levels, between 251 and 57,600 IU/L (with a normal range between 60 and 174 U/L) and elevated liver enzymes. The highest CPK level (57,000 IU/L) was identified in 3½-year-old patient with DMD phenotype (#1). Elevated serum CPK levels were also found in analyzed samples from the female patients. Level ranged between 1,300 and 4,900 IU/L.

The age of patients in the symptomatic female group (*n* = 4) ranged from 4 years (the twin sisters) to 9 years. Clinically, the manifestations of the twins (#F1/#F2) were different as follow #F1 was immobilized in a wheelchair, presented facies in a full moon, did not raise hands above the head, muscular hypotrophy, and generalized hypotonia predominantly in the lower limbs, pseudohypertrophy in the legs, bilateral, diminished muscle tone, and strength. #F2 presented normotonic and normokinetic muscular system, normal muscular strength, and independent walking were possible.

All muscle biopsies analyzed by routine histological staining (hematoxylin & eosin staining, modified Trichrome Gomori) showed the specific changes of muscular dystrophy: round shape muscle fibers, fiber size variation, the presence of necrotic and regenerating fibers, the presence of some atrophic and hypertrophic fibers, endomysial and perimysial connective tissue proliferation, and increased number of internal nuclei.

### Dystrophin Protein Screening

To determine the presence, absence, reduced expression, and localization of dystrophin in skeletal muscle samples, we used IF screening. WB was performed as an additional method to observe the total amount of dystrophin and the normal or reduced-sized of dystrophin in muscle fibers.

For a more accurate interpretation of the protein expression results, we used the following notation for the intensity of signal compared with the controls: normal intensity signal (+), reduced signal (+/−), very low signal (+/−), and absent signal (−). The controls (normal muscle) were worked simultaneously with the patients' samples.

Immunostaining analysis with anti-dystrophin antibodies for all three domains (C-terminus, rod domain, and N-terminus) allowed us to identify a variable pattern of dystrophin expression: (i) absence of staining for dystrophin (*n* = 14) for all three domains, (ii) a weak intensity staining (*n* = 12), (iii) irregular labeling with absence of one domain of dystrophin (*n* = 10); (iv) almost normal staining or slightly reduced (*n* = 4).

WB evaluation of dystrophin highlighted a complete lack of bands for dystrophin at 427 kDa (*n* = 30), a reduced intensity of dystrophin bands (*n* = 6) and several cases with differences between intensity of bands for different domains of dystrophin (*n* = 4) (no detectable band for one domain while the other showed a variable reduction).

As is known, a complete absence of dystrophin by WB leads to a DMD diagnosis while a variable expression of dystrophin was correlated with the BMD phenotype. Based on the expression pattern of dystrophin by quantitative method, the patients were classified into two groups: group 1 - DMD patients with the absence of dystrophin (30 cases), group 2 - BMD patients with dystrophin deficiency (10 cases).

**Figure 2:**
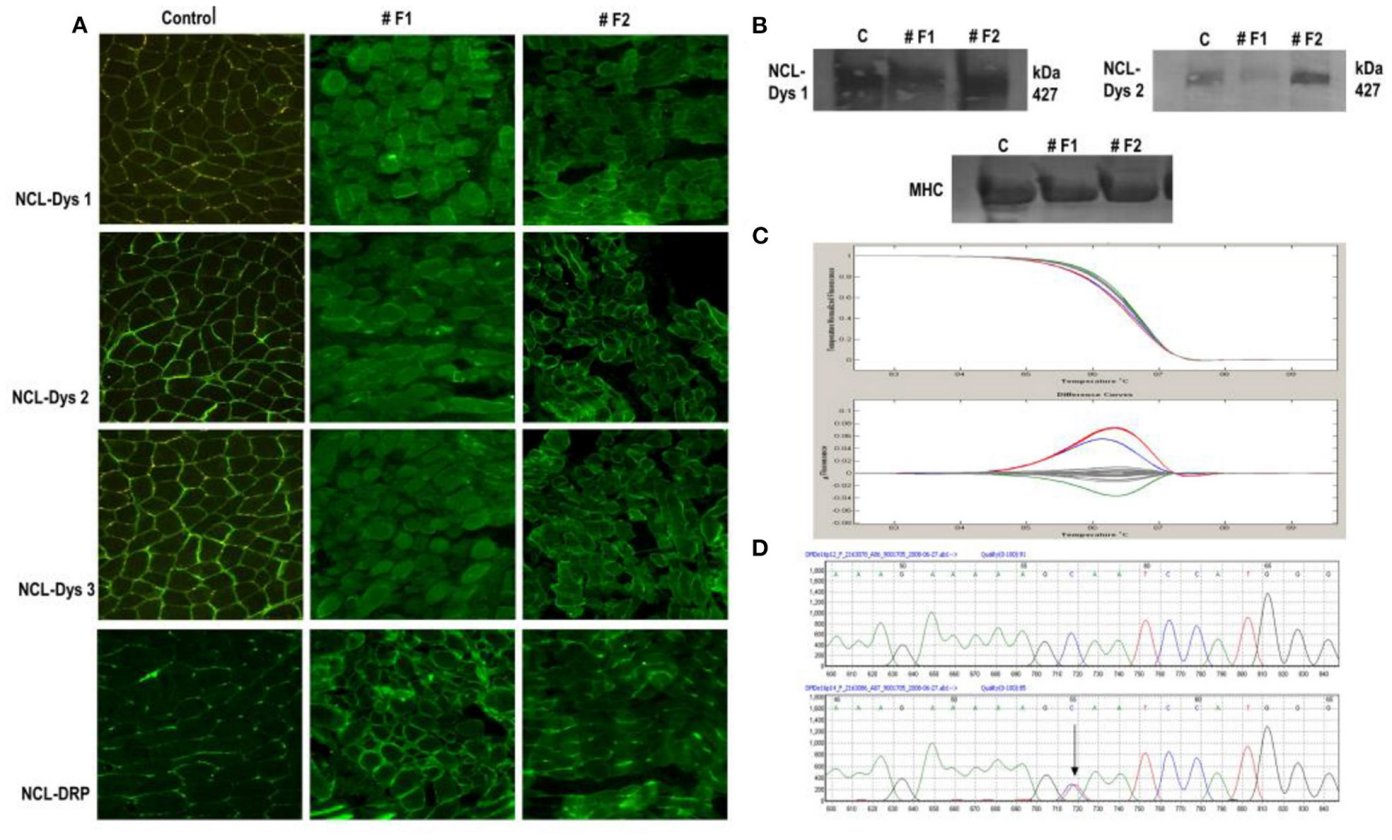
Immunofluorescent staining of dystrophin in normal control muscle and twin sisters (#F1 and #F2). **(A)** Immunostaining for dystrophin with antibodies against all three dystrophin domains for twin sisters revealed a difference between them. The first sister (F1) shows a reduction/ discontinuous signal for dystrophin and overexpression of utrophin from NMJ to the whole sarcolemma while the other sister (F2) presented only a slight reduction of signal for dystrophin. Scale bar 50, μm. **(B)** Dystrophin WB analysis of muscle biopsy sample from both sisters revealed the same difference between sisters with reduced intensity of bands for dystrophin with (a) antibody directed against the dystrophin rod domain (DYS1) and (b) antibody directed against the dystrophin C-terminal (DYS2) for sister F1 and an almost normal expression for sister F2 compared with control muscle sample. **(C)** HRM analysis revealed the same sequence variant for both sisters in exon 16 in the *DMD* gene (red line). Also, patient #18 was identified with a variation in exon 16 (blue line) compared with normal (Gray lines). **(D)** A section of the sequencing electropherogram for one twin compared with control.

In the case of the twin sisters, dystrophin expressions by IF showed differences between them for all three dystrophin antibodies used and for utrophin. One twin (#F1) showed a considerable reduction in the intensity of dystrophin staining, and overexpression of utrophin. Her twin sister (#F2) showed only a reduced intensity of the signal for dystrophin by IF and an almost normal expression of utrophin. By immunoblot, only very slight differences in the intensity of the bands between the two twins were observed. The first sister (#F1) presented a reduced intensity of bands for dystrophin, and the second sister (#F2) a normal expression of dystrophin was identified (Figure 2).

### Molecular Results

#### Deletions and Duplications Patterns in the *DMD* Gene

**Figure 3:**
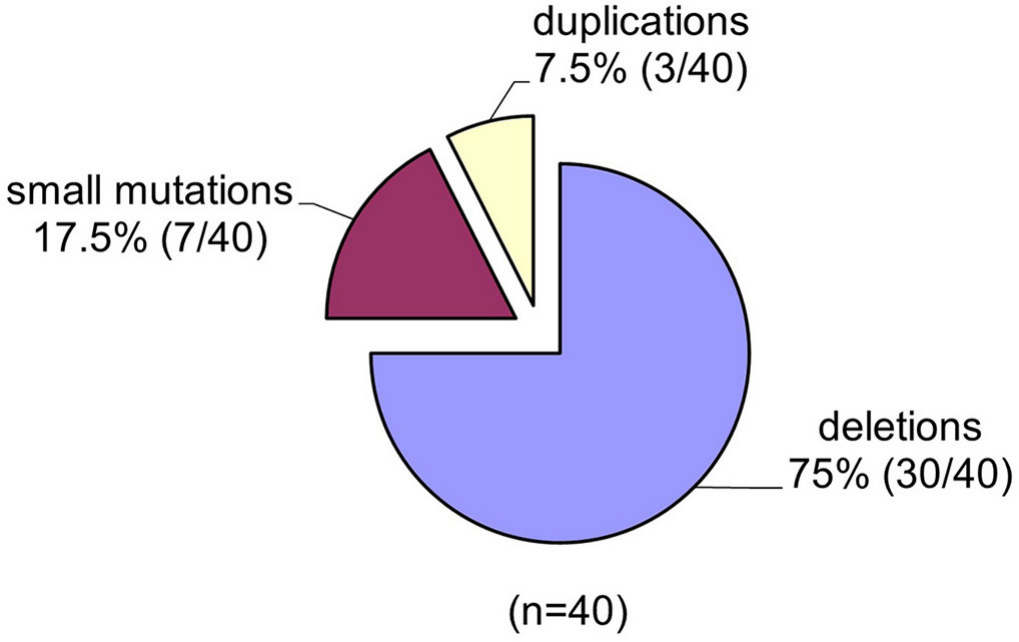
The spectrum of mutations identified in the *DMD* gene in the group of 40 unrelated Romanian patients.

Spectrums of deletions/duplications in all 79 exons of the *DMD* gene were determined by the MLPA technique (Table 1). Large mutational events were identified in 33 of the 40 male patients (Figure 3).

**Figure 4:**
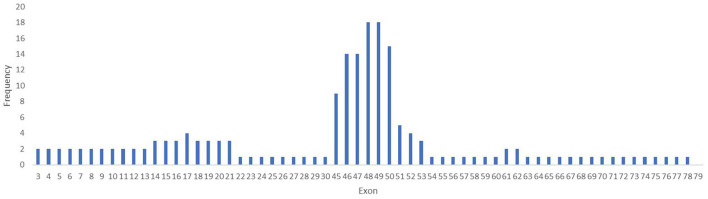
Frequency of mutation per exons in the *DMD* gene. The exons 48 and 49 are the most involved in mutations in the *DMD* gene in our study lot.

Within the 30 deletions identified in our study, 27 (93%) were located at the distal hotspot region of the *DMD* gene and involved exons 44–55, while two mutations (6.6%) were located at the proximal hot spot region that includes exons 2–19. Both deletions identified at the proximal hot spot region began within the N-terminal domain with exon 3. For patient #6, the deletion extended to exon 21 in rod domain and for patient #7 to exon 30. One deletion was identified outside the hotspot regions and included exons 53–62. No deletion was found between exons 63–79. The most frequent exons involved in a deletion were exons 48 and 49, both identified in 18 cases (Figure 4).

Multiple exon deletions were identified between exons 48–50 (6/30, 20%), followed by exons 45–50 (3/30, 10%), exons 45–47 (3/30, 10%), and exons 48–49 (3/30, 10%). Also, deletions of exons 46–47 and 46–48 were identified in two samples (2/30, 6.6%). Most large deletions spanned between one to 10 exons in 28/30 cases (93.33%), 11–20 exons in 1/30 (3.33%), and 21–30 exons in 1/30 (3.33%). Deletions that involved three exons were most common being identified in 10 cases (33.33%) followed by deletions that involved two exons (6/30, 20%), and deletions of four exons (4/30, 13.33%).

The MLPA method allows the identification of single exon involvement in a deletion in two out of 30 cases (6.6%) (exon 45 and 51). Both mutations were confirmed by a total absence of dystrophin on WB and PCR amplification with specific primers flanking exon 45 and 51.

All identified deletions were checked using the frame-shift checker from the Leiden Muscular Dystrophy website (www.dmd.nl) to establish whether the mutations are disrupting or non-disrupting with respect to the reading frame. The molecular alterations identified in 21 male patients were out-of-frame, and according to the reading frame rule, determine the absence of dystrophin from muscle and a DMD phenotype. Nine patients presented in-frame deletions which permitted the generation of a shorter, but functional dystrophin protein that is associated with a BMD phenotype.

Only three duplications were identified in our samples. Patient #23 presented a duplication of a large fragment involving 18 exons (16 to 78) that started at the 3′ end of the gene encoding for the C-terminal domain of dystrophin. This resulted in a less severe BMD phenotype. Two patients (#22 and #40) were identified with duplications that involved small fragments located in the rod domain of the *DMD* gene. Both duplications were out of frames which alters the reading frame of dystrophin mRNA and gives rise to the DMD phenotype. One duplication (#40) encompassed five exons from 17 to 21 and the second one (#22) involved four exons from 14 to 17. All identified duplications respected the reading frame theory and correlated well with dystrophin expression.

No large mutations were identified by MLPA in the symptomatic and asymptomatic female group.

#### Small/Point Mutations

Since there are no point mutation hotspots reported in the *DMD* gene, searching for sequence variations requires amplification of all 79 exons and eight promoters. Due to the limited amount of samples, time-consuming, and the expensive costs for searching for small mutations in this huge gene, we randomly choose 40 exons for analysis. Screening for point mutations was performed in all non-deletions/duplications patients (*n* = 7), and additionally in the female group.

Here, we report nine different changes found in our patients (male and female) including two non-sense, two missense, two frameshift, two splicing, and one silent sequence variant. The results are shown in Table 2. We did not detect any sequence variants in the following exons: 1, 3, 4, 5, 6, 8, 9, 10, 11, 12, 22, 23a, 24, 27, 28, 29, 31, 36, 38, 39, 40, 46, 50, 53b, 58, 59, 60, 62, 65, 70, 73, 74, and 76. All exons which showed modification in the melting curve compared with the wild-type profile, were sequenced.

**Table 2:** Variants detected by Sanger sequencing in analyzed samples.

**Case ID**	**Exon ID**	**Nucléotide change**	**Mutation type**	**Dystrophin protein change**	**Dystrophin protein domain**	**Clinical signifiance**	**References**
**Male**							
18	Exon 16	c.1990C>T	Non-sense	p. (Gln664*)	Rod-domain	Pathogenic	(17)
19	Exon 57	c.8507G>T	Missense	p. (Gly2836Val)	Rod-domain	Unknown pathogenity	Novel
21		Not detected					
28	Exon 23	c. 3021del	Frameshift	p. (Lys1008Argfs*36)	Rod-domain	Unknown significance	Novel
33	Exon 53	c.7728T>C	Silent mutation	p. (Asn2576Asn)	Rod-domain	Likely benign	(24)
34	Exon 65	c.9563_9563+1 insAGCATGTTTA TGATACAGCA	Frameshift	p. (Gly3189Alafs*4)	Rod-domain	Probably damaging	Novel
37		Not detected					
**Female**							
F1/F2 Twin sisters	Exon 16	c.1843C>T	Nonsense	p. (Gln615*)	Rod-domain	Pathogenic	(25, 26)
F3	Intron 78	c.11046+119 A>G	Splicing	p.?	C-terminal	Benign	(17, 27)
F4	Intron 52	c.7661-60T>A	Splicing	p.?	Rod-domain	Unknown pathogenity	Novel
F5 Twin mother	Exon 59	c.8762 A>G	Missense	p. (His2921Arg)	Rod-domain	Benign	(28–31)
F6 patient' sister #1	Exon 53	c.7728T>C	Silent mutation	p. (Asn2576Asn)	Rod-domain	Benign	(24)

No sequence variants were found in the analyzed exons from two patients with dystrophin deficiency. This could have been because of the limited number of exons investigated, or because the mutation was present in an intron or in a regulatory region (23).

Generally, the sequence variants identified in our study groups were located in the rod domain, especially around the distal hot spot region of the *DMD* gene. An only one-point variant was located within exon 78, in the C-terminal domain of dystrophin. All mutations identified in male patients were accompanied by the total absence of dystrophin in the muscle tissue on WB and indicate a DMD phenotype.

**Figure 5:**
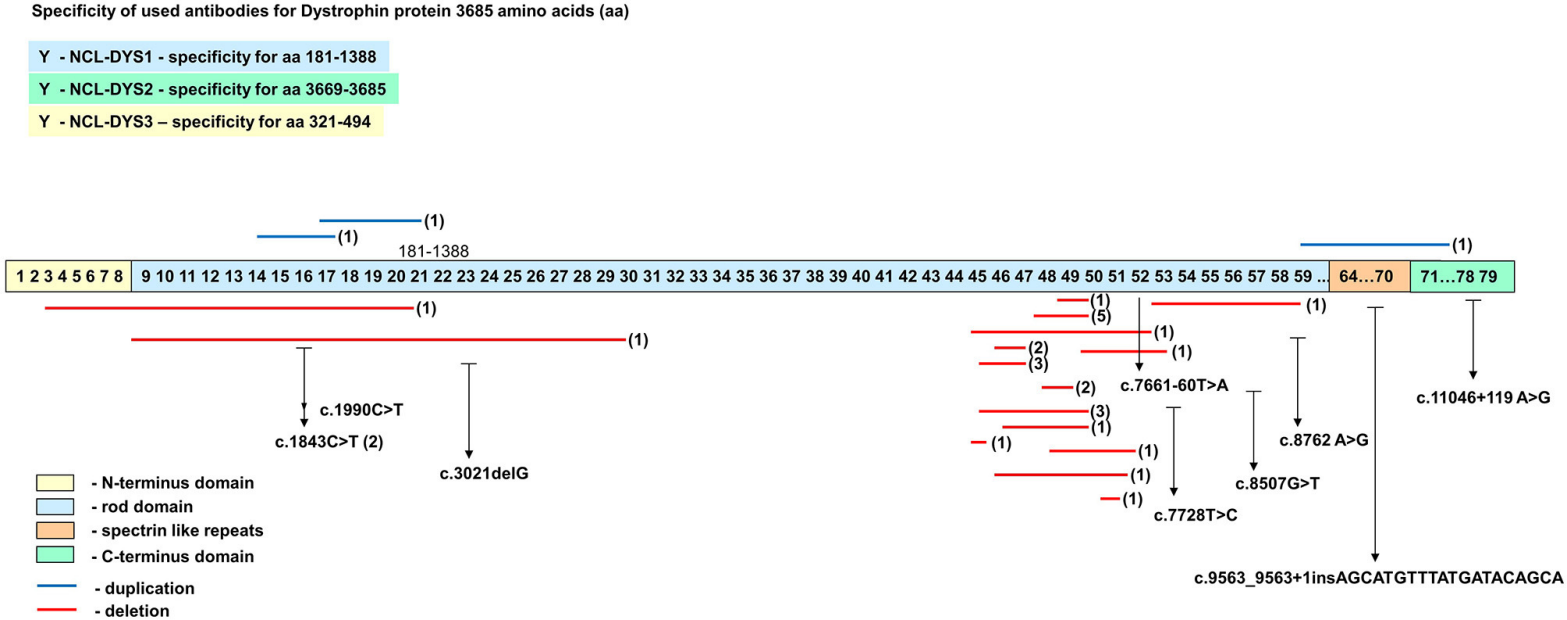
Schematic representation of the distribution pattern of mutations along the *DMD* gene.

The distribution of all mutation identified in the *DMD* gene is presented in Figure 5. Based on the mutational pattern of the *DMD* gene, 23 patients had a DMD phenotype, and 10 patients had a BMD phenotype.

## Discussion

To our knowledge, this is the first study describing the correlation of protein data with genetic characterization in DMD/BMD patients from Romania. Using immunohistochemistry and WB for the study of dystrophin protein and molecular genetic testing methods (MLPA, HRM, and sequencing) for the *DMD* gene analysis, we investigated the type, size and location of dystrophin mutations distribution that occur in the *DMD* gene and how these mutations affected the dystrophin protein expression in our cohort.

Protein analysis was the first step in our workflow. Dystrophin protein expression was assessed in all 40 patients from the study group. All patients showed total or partial protein deficiency (32–34). The total absence of protein in 14 patients was confirmed by quantitative and qualitative methods. Moreover, in 17 patients who showed a low level of dystrophin signal on cryosections by IF, showed a total absence of dystrophin bands by the WB method Based on western blot results dystrophin levels for the BMD patients ranged from 9 to 80%, when detected by the rod domain antibody and from 7 to 68% when detected by the C-terminal antibody.

These sizeable differences between results obtained by these two methods suggest a greater sensitivity of the WB method compared with IF (33). However, determining the most accurate level of muscle dystrophin by immunoflorescence requires automatic image capture and analysis, knowing that the acquisition factor can negatively influence the measurement of fluorescence intensity.

All samples which displayed an absence of signal for dystrophin on WB were characterized as DMD phenotype while samples that displayed a reduced signal for dystrophin were identified as the BMD phenotype (35, 36). Based only on protein analysis, 31 cases with DMD and nine cases with BMD were identified.

The availability of immunohistochemical investigation results allowed us to demonstrate the pathogenic effect of a different mutation that occurred in the *DMD* gene on protein expression and to identify two cases with traces of dystrophin. One of the samples, case #36, presented a few fibers with the low signal by IF in the N-terminal domain. This was accompanied by a deletion in the mid-distal region of dystrophin that involved exons 48–52 and resulted in a shift of the translational reading frame. The other patient, case #10, presented few fibers with traces of dystrophin in rod domain, was identified with an in-frame deletion of exons 49–53. In our cases, traces of dystrophin were observed in few fibers of patients that carry-out both out-of-frames and in-frame mutations but who give rise to the DMD phenotype. The functional effect of traces of dystrophin is still unknown, but it is suggested that dystrophin can be produced by a different mechanism, hinting at a possible prognostic value and an important tool for a further exon skipping therapy (37) as well as for prognosis of disease severity (38, 39). It is worth noting that in both these patients, traces of dystrophin were observed only by IF while WB analysis showed a total absence of signal for anti-dystrophin antibodies, probably because of the very small amount of dystrophin produced.

### Protein Data and Molecular Correlation

The correlation between protein and genetic data results permitted us to compare the diagnoses predicted by protein analysis alone with that obtained by molecular analysis. This enabled us to check the applicability of the frameshift theory and to identify exceptions from the reading frame rule. Our immunohistochemical findings and exonic deletions were not in accordance with two cases which mean a fit of the reading frame theory in 95% of 40 Romanian patients with dystrophinopathies. Such exceptions from the reading frame rule have also been reported by other researchers (40–44).

### Exceptions to the Reading Frame Rule

The first exception identified in our study group is patient #7, with an out-of-frame mutation (del ex 53–62) which disturbed the reading frame and determined a severe DMD phenotype. Protein analysis showed only a reduced level of dystrophin in the muscle (63% detected by the rod domain antibody and 36% detected by the C-terminal antibody) which correlates with a BMD phenotype. One possible explanation for a less severe phenotype is that even if it is an out-of-frame mutation that involved exons 53–62 and affected hinge IV region, the WW domain and EF-hands motif of this region is maintained, thus permitting binding of carboxy-terminus of β-dystroglycan by which the dystrophin is anchored at the sarcolemmal (45). Also, patient #10 presented a residual dystrophin expression in only a few fibers from rod domain by IF, a total absence of dystrophin by WB, and correlation with an in-frame deletion of exons 49–53 giving rise to a BMD phenotype. Nevertheless, the absence of dystrophin by WB suggests a DMD phenotype rather than a BMD, since a DMD diagnosis is demonstrated by the absence of dystrophin (46). This deletion involved spectrin-like repeats R19-20 that flanking hinge III region of dystrophin. This had been shown that is important for the dystrophin localization as well as for proper stability of protein structure (47–49). Therefore, the exact role of hinge regions is still unknown, but it appears that these regions play an important role in the proper functioning of dystrophin and requires further investigation. Future studies of cases that do not respect the reading frame rule will be useful in investigating the correlation between the variability of clinical features and specific dystrophin alteration.

Frameshift mutations which involved deletion of exon 51 (patient #9), exons 49–50 (patient #27), and exon 45 (patient #25) were previously reported as BMD phenotypes (50). Establishing the exact phenotype for these patients should be done with caution and with consideration of dystrophin protein expression rather than based only on gene mutations pattern.

Our DMD patients with out-of-frame mutation had a CPK levels up to 300 times higher upper limit of normal and no dystrophin expression was found. Patients with BMD have a CPK level up to 20 time's higher than the maximum reference range and carrier female up to eight times higher. We also identified one DMD patients (#37) with normal CPK level. In agreement with data published by other researchers, this may be due to a serious muscle damage considering the almost total absence of dystrophin in muscle. Normal concentrations of serum CPK have been also reported in DMD-associated dilated cardiomyopathy (DCM).

### Variability of Dystrophin Expression in Patients With the Same Deletion

An interesting observation in our samples was the variability of the dystrophin expression by IF in patients with the same deletion (e.g., exons 48–50) with the age of onset between 3 and 11 years. For instance, patients #1, #2, and #5 showed a total absence of dystrophin, while patients #3 and #4 revealed a slowly reduced level of dystrophin expression for all domains of dystrophin. There are some reports of differences in phenotype in patients that share the same deletion. This could be explained by different intronic breakpoints that alter translation efficiency (34, 35). Differential stability of mutated dystrophin (51, 52) as well as different pathogenic mechanisms could contribute to this phenotype variability.

### Deletions in *DMD* Gene

The MLPA technique enabled us to assess all large mutations in the *DMD* gene and the results indicate a higher incidence of deletions in our study group 75% (30/40) and a lower incidence of duplications 3/40 (7, 5%). The frequency of large mutations in the *DMD* gene varies in different populations worldwide. While American studies report an incidence of intragenic deletions of around 60% (53), European studies report an incidence of deletions of between 51% in Denmark (54) and 90% in the Netherland (55). Our results indicate an incidence of deletions of 75%, closer to reports from Italy (72%) (56), and Hungary (73%) (57). These differences between reported deletion rates could be due to several factors such as the number of patients included in the study, selection criteria as well as demographics features.

### Duplications

The frequency of duplications in our study was reduced (3/40). The duplications identified caused severe DMD phenotype being associated with out-of-frame mutations in the rod domain, absence of dystrophin, and a higher level of CPK for two patients. Duplication of 19 exons (dup 61–78) in the C-terminal domain (patient #23) was associated with an in-frame mutation, a reduced level of dystrophin, and a BMD phenotype.

### Hotspot Mutation Regions

Previous reports have demonstrated the presence of two hot-spot mutation regions (58, 59) for deletion and duplication in the distal part of the *DMD* gene between exons 44–55 and the proximal part between exons 2–10 (60). These regions, reported to have a higher incidence of mutations, are required for a proper function of dystrophin providing stretching and flexibility of proteins through interaction with actin (61). Our results confirmed the presence of the two-known hot-spot regions with a higher frequency of mutations. Also, there was observed a greater tendency for mutations in the distal hotspot region of the *DMD* gene (93%) from the central rod domain of dystrophin rather than a minor region, in which only four mutations (6.6%) were located at the proximal hot spot region.

### Small/Point Mutations

Point mutations identified in the rod domain of male patients were correlated with the absence of dystrophin (Table 2). Although the presence of hot spots in the *DMD* gene for point mutations has not been reported so far, we identified a higher frequency of mutations in exon 53 in samples without a family relationship. Between them, one male patient #33 and one female #F6 shared the same mutation c.7728T>C. Female patient #F4 also presented a substitution that affect exon 53 (c.7661-60T>A).

Exon 16 was identified with a mutation in three samples: both twin sisters that share the same mutation in position c.1843C>T and patient #18 who presented mutation c.1990C>T. Even if both twin sisters shared the same mutation c.1843C>T in exon 16, the difference in phenotype was obvious regarding the clinical features and dystrophin expression. The different phenotypes between sisters could be explained by the inactivation of a normal X-chromosome in a large cell mass in one sister compared with the other (62). The twinning event occurs after X-chromosome inactivation (63). The twins' mother carried a missense substitution within exon 59, c.8762 A>G (p. His2921Arg). This mutation was previously reported as a polymorphism (24, 29, 30) and it was considered not to affect dystrophin function (23, 31). Because the mother has only a benign variant in the *DMD* gene, she can be considered as a germline mosaic case for the pathogenic variant of the twins.

Of the female relatives (mother, sister, and two primary cousins) of patient #1 (del 48–50), only the sister presented a silent substitution in exon 53 which changes T to C in position c.7728T>C, DB-ID: DMD_00763 (p. Asn2576Asn). This variant does not alter the protein sequence and is considered a benign variant. The presence of two different mutations in the *DMD* gene in one family could be explained by the presence of germline mosaicism in the mother or by parental origin.

Three sequence variants associated with DMD phenotype (c.8507G>T, c.3021delG and c.9563_9563+1insAGCATGTTTATGATACAGCA) identify in male patients and one (c.7661-60T>A) identified in the symptomatic female have not been reported so far, according to the OMIM database and Leiden Muscular Dystrophy Database.

### Correlations Between Type, Location, and Size of the Mutation in the *DMD* Gene, and the Severity of Phenotype

Although the relationships between DNA mutation and the protein profile are difficult to determine, the genotype-phenotype correlation seems to be the key to understanding the mechanism of this disease. With the strategy employed in this work, some conclusions have been drawn regarding the correlations between type, location, and size of the mutation in the *DMD* gene, and the severity of phenotype.

In our study group, we observed that disease severity is influenced by the type of mutation and depends on whether the mutation maintained or not the reading frame. A severe DMD phenotype is usually caused by an out-of-frame mutation that determines the absence of dystrophin in muscle while a less severe BMD phenotype is determined by an in-frame mutation that affects the level of dystrophin in muscle. In 95% of our cases, the correct phenotype could be predicted by the reading frame theory.

In accordance with previous studies, the changes of a single base in the DNA alters the reading frame of dystrophin mRNA and introduces a stop codon. This in turn determines the premature termination of protein translation (64, 65) and causes a DMD phenotype as we have shown for cases #18 and #19. Also, insertion of a small fragment of 20 bp (AGCATGTTTATGATACAGCA) in an exon generated a DMD phenotype (case#34).

Our data results, together with previous studies provide evidence that some regions of the *DMD* gene are critical for dystrophin stability (66), and for maintaining the interactions between the extracellular matrix, and subsarcolemmal cytoskeleton, *via* glycoprotein complex (67). The mutations that affect these regions of the dystrophin gene have a major impact on protein expression in muscle and implicitly on phenotype severity.

We observed that most of the large mutations, as well as sequence variants identified, were located in the rod domain of the dystrophin (87%) and correlate with both DMD and BMD phenotypes. This finding suggests the importance of the rod domain and hinge region for the proper functioning of the muscle. Future studies are required to understand the role of the rod domain in dystrophin function. In this study, it appears that the size of the mutation does not influence the severity of the phenotype.

Studying the *DMD* gene is challenging because of the large size of the gene, the large variety of mutations, and the difficulty with the genotype-phenotype association. Gene size and mutation variety mean that improving genetic tools for complete sequence analysis of the *DMD* gene has become a necessity. Next-generation sequencing (NGS) has become the tool for the entire gene investigation. It provides detailed information on the mutational events occurring at the gene level. However, the high cost of the latest equipment means that this new method to be used in only a few laboratories. Currently, the MLPA technique (68) is considered the most powerful minimally invasive technology for accurate detection of gross rearrangements in all 79 exons of the *DMD* gene in males as well as in females. In combination with high-throughput HRM molecular technique becomes an important screening tool for detecting complex in the *DMD* gene. Sequencing only exons with abnormal melting profiles allow accurate clarification of the exact genotype of the patients and reduces cost and time spent identifying small mutations in this enormous gene.

Many promising therapeutics strategies like exon skipping with antisense oligonucleotides (AOs) (69–71), protein up-regulation (72), and stem cell transplant (73) are under development and aim to restore the reading frame, and in turn, dystrophin production in skeletal muscle. All these therapeutic approaches rely on exact knowledge of the molecular and protein profile of the patients and are a requirement for the enrolment of the patients in clinical trials. However, for a correct diagnosis, and to benefit from the newest genetics' therapies, only gene and protein analysis provide valuable data for both diagnosis and research. For the carrier status of women at risk, genetic diagnosis is of particular value in prenatal counseling, knowing that type and localization of a mutation could anticipate the severity of the disease. Taking into consideration the lack of effective treatment for these diseases, the implementation of prenatal diagnosis and genetic counseling could reduce the risk of having an affected child.

## Conclusion

The strategy used in this study of integrating protein analysis with DNA molecular analysis achieved the following: (i) clarified the exact molecular and cellular profile of DMD patients, (ii) provided information about the mutational spectrum in the Romanian population, (iii) identified cases with discordant phenotype, (iv) shows the importance of protein and genetic data correlation for the correct identification of the phenotype, (v) identified, characterized, and contributed to variant databases with four novel point mutations in the rod domain of dystrophin-associated with the disease. In conclusion, our study provides a strategy for a complete characterization of patients with dystrophinopathy useful in selecting those cases that may benefit from targeted gene therapies.

## Data Availability Statement

The datasets presented in this study can be found in online repositories. The names of the repository/repositories and accession number(s) can be found in the article/Supplementary Material.

## Ethics Statement

The studies involving human participants were reviewed and approved by University of Bucharest; Colentina Clinical Hospital. The study was approved by the Ethics Committees of the institutions by a contractual agreement between them. Written informed consent to participate in this study was provided by the participants' legal guardian/next of kin.

## Author Contributions

GG: conceptualization and prepared the manuscript. GG and RV: the experimental work and analysis of data. EM: evaluated the histopathology of snap frozen muscle tissue. DP: a medical doctor with experience in neuromuscular disorders and performed clinical evaluations on patients. GG, EM, RV, DP, and EI: critically review of the manuscript. All authors read and approved the final version of the work.

## Funding

Funded by grants from Ministry of Research and Innovation in Romania, under Program 1—The Improvement of the National System of Research and Development, Subprogram 1.2—Institutional Excellence—Projects of Excellence Funding in RDI, Contract No. 7PFE/16.10.2018; CEEX 40/2005; National Program 31N/2016/PN 16.22.02.05 and 1N/2019/PN19.29.01.03 and PN 19.29.02.01.

## Conflict of Interest

The authors declare that the research was conducted in the absence of any commercial or financial relationships that could be construed as a potential conflict of interest.

## Publisher's Note

All claims expressed in this article are solely those of the authors and do not necessarily represent those of their affiliated organizations, or those of the publisher, the editors and the reviewers. Any product that may be evaluated in this article, or claim that may be made by its manufacturer, is not guaranteed or endorsed by the publisher.

## Footnotes

^1^Available from. www.dmd.nl/http://www.dmd.nl/database.html
