# Supplementary material for: Combining Protein Expression and Molecular Data Improves Mutation Characterization of Dystrophinopathies
Source: Front Neurol. 2021 Dec 7;12:718396. doi: 10.3389/fneur.2021.718396 (PMC8689184; doi:10.3389/fneur.2021.718396)
Supplement: Supplementary file 1 [file Data_Sheet_1.docx]

NCBI Reference Sequence: NM_004006.2. Homo sapiens dystrophin (DMD), RefSeqGene (LRG_199) on chromosome X

| **Mutation** | **Mutation Database in which the mutation was reported** |
| --- | --- |
| Variant #0000813040 (NC_000023.10:g.32486756del, DMD(NM_004006.2):c.3021del) | https://databases.lovd.nl/shared/variants/0000813040#00000024 |
|  |  |
